# Automatic XAFS measurement system developed at BL14B2 in SPring-8

**DOI:** 10.1107/S0909049511042518

**Published:** 2011-11-15

**Authors:** Hiroshi Oji, Yosuke Taniguchi, Sayaka Hirayama, Hironori Ofuchi, Masashi Takagaki, Tetsuo Honma

**Affiliations:** a Japan Synchrotron Radiation Research Institute (JASRI), 1-1-1 Kouto, Sayo-cho, Sayo-gun, Hyogo 679-5198, Japan; b SPring-8 Service Co. Ltd, 2-23-1 Kouto, Kamigori-cho, Ako-gun, Hyogo 678-1205, Japan

**Keywords:** XAFS, automation

## Abstract

A novel XAFS measurement system has been developed in which XAFS measurements can be performed including sample-loading and detector adjustments. With this system, XAFS measurements of up to 80 samples in both transmission and fluorescence modes can be carried out. The adjustment of the optical components has also been automated. It not only saves manpower and measurement time, but also improves the accuracy and reliability of sample alignments.

## Abstract 2

A novel automatic measurement system has been developed, in which the XAFS measurement of up to 80 samples in transmission and fluorescence modes can be carried out.

## 1. Introduction

Nowadays, X-ray absorption fine structure (XAFS) spectroscopy is one of the popular analysis tools for the ordinary scientific and engineering researcher. In particular, this technique is now indispensable for new energy and environmental fields such as rechargeable batteries, fuel cells, chemical catalysts, *etc*. In such types of research, high-throughput measurements are often largely expected because a large number of samples with different compositions and/or synthetic conditions should be measured. Since the recent development of XAFS measurements in continuous mode (QXAFS; Frahm, 1988 ▶; Uruga *et al.*, 1999 ▶, 2009 ▶), the speed of the measurement itself has been dramatically improved. However, operations accompanied by XAFS measurements, such as sample loading, sample alignment, detector settings, input of measurement conditions, *etc.*, are usually performed manually, which has been the major bottleneck in the XAFS measurement lately.

In the present paper we will describe a novel automatic XAFS measurement system developed at BL14B2, one of the public beamlines dedicated for engineering science research in SPring-8 (Honma *et al.*, 2010 ▶). XAFS measurements of up to 80 samples, including the above-mentioned operations, can be performed automatically by this system, which can be operated in both transmission and fluorescence modes. The optical adjustment procedure and the flow of the appropriate gas for ion chambers have also been automated. As part of the experimental equipment at BL14B2, a brief summary of this system has already been reported elsewhere (Honma *et al.*, 2010 ▶). Here we will focus on the automatic XAFS measurement system in more detail, which has recently also become available for the fluorescence mode.

## 2. Instrumentation

### 2.1. Overview

**Figure 1:**
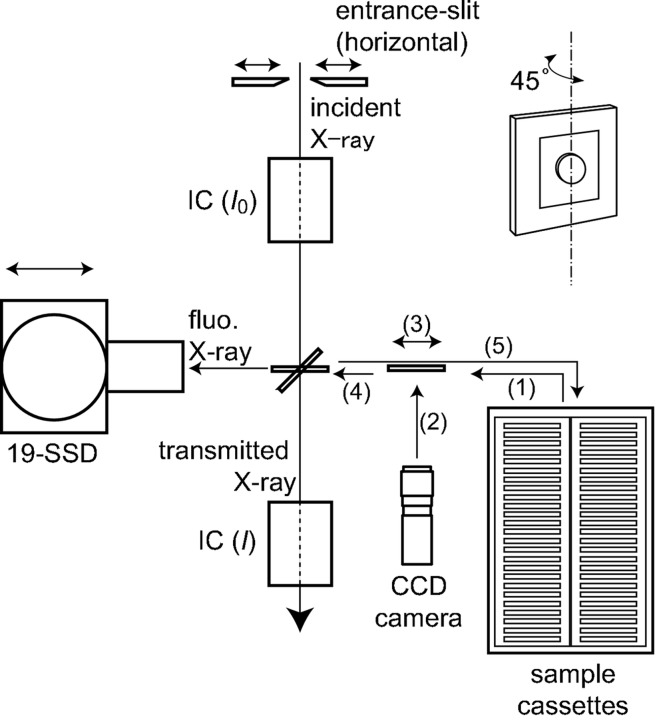
Schematic illustration of the sample-loading and XAFS measurement system.

Fig. 1 ▶ shows a schematic illustration of the automatic XAFS measurement system, in which the automatic sample-loading system has been integrated with the conventional XAFS measurement system. A sample is picked up from a sample cassette and is inserted into the X-ray path by the sample-loading system. In the case of fluorescence-mode measurements, the sample is rotated by 45°. Details of the sample alignment will be described in §2.3. The intensities of the incident and transmitted X-rays (*I*_0_ and *I*_1_, respectively) are monitored by two ion chambers in transmission-mode measurements. A 19-element Ge solid-state detector (19-SSD) is placed at the side of the sample for the fluorescence mode. The distance from the sample to the 19-SSD can be adjusted using a motorized linear stage beneath. The intensity of the incident X-rays can be adjusted using an entrance slit.

**Figure 2:**
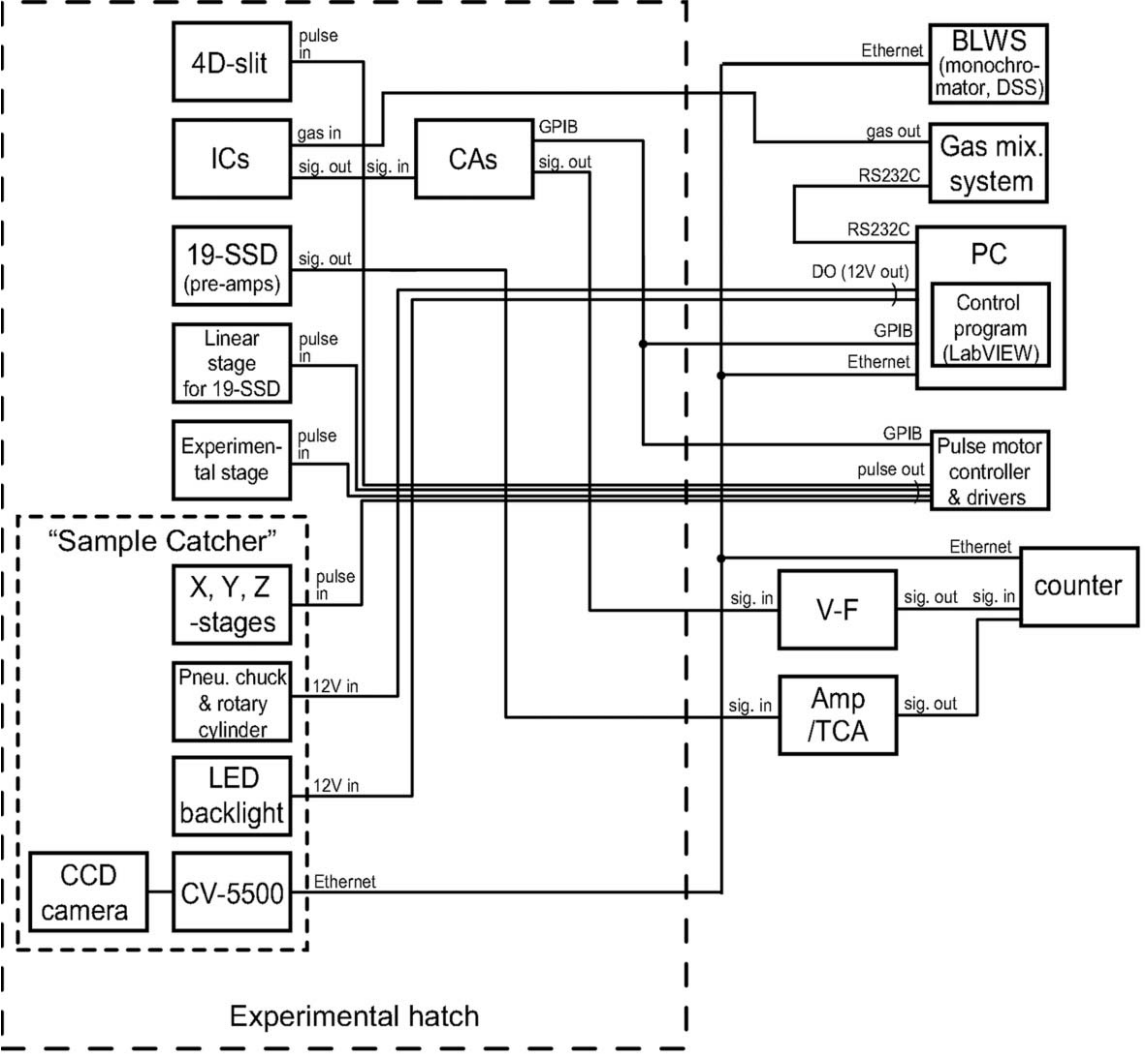
Diagram of the control and data acquisition system of the automatic XAFS measurement system.

A diagram of the control and data acquisition in the automatic XAFS measurement system is shown in Fig. 2 ▶. The ion currents from the ion chambers are amplified by current amplifiers and then digitized by voltage-to-frequency (V–F) converters. The fluorescence signal collected by 19-SSD is amplified and processed by pre-amplifier and amplifier-TCA (three-channel analyzer) modules. These data are accumulated in high-speed counter units. All of the motorized stages and the entrance slit are driven by pulse motor controller and drivers. The gases for the ion chambers are supplied from a gas distribution system with 16 mass flow controllers (HORIBA STEC, PAC-6C). The monochromator, slits, mirrors and downstream shutter (DSS) are controlled by the beamline workstation (BLWS). The instruments are controlled by PC *via* GPIB, TCP/IP, RS232C or the signal from a digital output (DO) card.

The automatic XAFS measurement system was developed in three steps. Firstly, a sample-loading system was solely developed. Then, the automatic XAFS measurement system for transmission mode was developed by integrating the sample-loading system and the existing measurement systems for XAFS. Finally, the system was improved to be also applicable to fluorescence-mode measurements.

An outline of the automatic optical adjustment system will be given in the following section (§2.2). Then detailed explanations of the sample-loading system and the automatic XAFS measurement system will be presented in §2.3 and §2.4, respectively.

### 2.2. Automatic optical adjustment system (‘Auto-Optics’)

The beamline optics and the gases in the ionization chambers can be adjusted automatically by the optical adjustment system described by Honma *et al.* (2010 ▶), which is now called ‘Auto-Optics’. The system of Auto-Optics has been programmed in the *LabVIEW* programming language. For the X-ray optics in BL14B2 and the front panel of Auto-Optics, see Figs. 1 and Fig. 2 of Honma *et al.*

If once the absorption edge and the net plane of the monochromator crystals [Si (111) or Si (311)] are selected and Auto-Optics is started, the adjustment will be made fully automatically. At first, one of the gases (He, N_2_, Ar or Kr) or a suitable mixture of these gases appropriate for the absorption edge tabulated in the program starts to flow from the gas-distribution system to the ionization chambers. If necessary, the net plane is changed by rotating the crystals around the 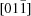 axis in the next step. Then the monochromator is fine-tuned by the rocking-curve measurement at the Bragg angle of the absorption edge designated above. Then, the positions of the optical components downstream from the monochromator are adjusted by scanning them in the following sequence: the vertical position of the first mirror, that of the second mirror, that of the slit right after the monochromator, the height of the experimental stage, and the horizontal position of the slit in the experimental hutch. Note that the glancing angle of the mirrors appropriate for rejecting the higher harmonics is calculated by an empirical formula. Each scan (a curve of *I*_0_ intensity *versus* the position) is analyzed by Auto-Optics, and the component is moved to an appropriate position automatically.

### 2.3. Sample loading system (‘Sample Catcher’)

Here we describe the details of our newly designed sample-loading system nicknamed ‘Sample Catcher’. The sample manipulator consists of a pneumatic chuck to grab a sample, a pneumatic rotary cylinder to rotate the sample by 45° to apply the system for both transmission- and fluorescence-mode measurements, and three motorized stages [SIGMA-KOKI, SGSP26-100(Z), SGSP26-200(X) and SGSP46-300(X)] to translate the sample three-dimensionally. Each sample, *e.g.* a pellet, is sealed by a transparent film and mounted in a sample holder of size 50 mm × 50 mm × 1–2.5 mm, which is compatible with film mounts commercially obtainable. Then, the sample holders are loaded into sample cassettes, in which up to 40 samples can be stored per cassette. Two cassettes can be set in the system, thus up to 80 samples can be loaded in a batch measurement.

Sample alignment in transmission mode is performed as illustrated in Fig. 1 ▶. At first, (1) a sample is picked up from a sample cassette and moved to the front of the CCD camera. Next, (2) the sample image (silhouette) is captured by the camera, and the pixel position of its centre of gravity is analyzed by a KEYENCE CV-5500 vision system. Then, (3) the position of the sample is adjusted so that the centre coincides with the reference pixel position determined beforehand with respect to the X-ray beam as described later. Finally, (4) the sample is moved to the measurement position. After the measurement, (5) the sample is returned to a sample cassette, and another sample for the next measurement is picked up. This procedure is repeated until the measurements of all samples are finished in a batch measurement.

The reference pixel position is determined as follows. Firstly, a metal plate with a pinhole of diameter 1 mm is scanned horizontally and vertically near the X-ray beam. From the profiles of the transmitted X-ray intensity during the scans, the position of the plate where the centre of the pinhole coincides with that of the X-ray beam is determined. Secondly, the plate is moved horizontally to the front of the CCD camera by a given distance (*e.g.* 70 mm), and the image of the pinhole is captured. Finally, the pixel position of the centre of the pinhole is analyzed by CV-5500, which becomes the reference pixel position for sample alignment. Thus if the sample is moved to the X-ray beam side by the given distance at step (4) as described earlier, the centre of the sample is on the X-ray beam in principle.

**Figure 3:**
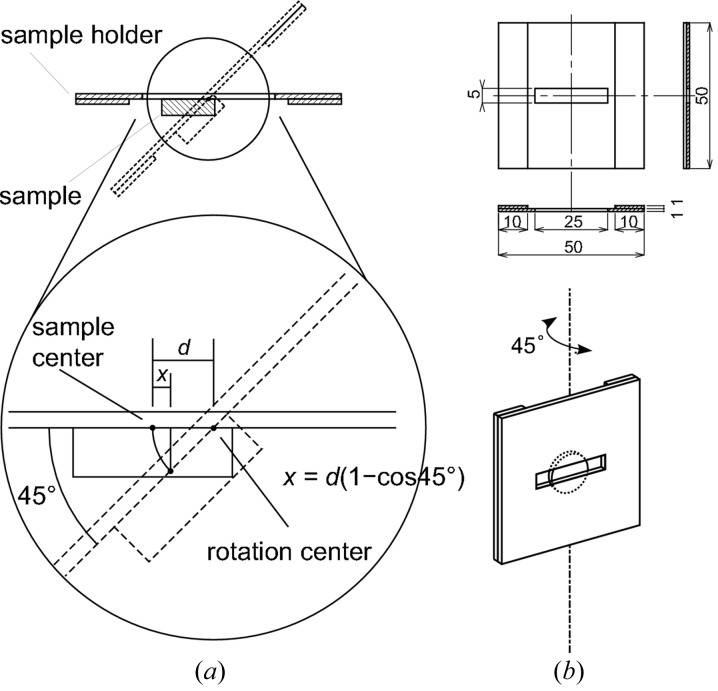
(*a*) Sample alignment in the case of fluorescence mode. (*b*) The special sample holder designed for fluorescence-mode measurements.

The sample alignment for fluorescence mode is slightly different from that for transmission mode, since the sample is rotated by 45° in fluorescence mode. The horizontal position of the centre of the sample surface deviates by sample rotation unless the centre of the sample surface is in the rotation axis. However, if the rotation axis is in the plane including the sample surface, and if the distance between the centre of the sample surface and the rotation axis (*d*) is known, the deviation in horizontal position by sample rotation (*x*) can be calculated as
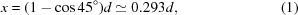
as illustrated in Fig. 3(*a*) ▶. To meet this condition, we use the special sample holder shown in Fig. 3(*b*) ▶ for the fluorescence-mode measurements. The first part of the sample loading in the fluorescence mode is the same as steps (1)–(3) in transmission mode. In step (3), the distance *d* can be estimated as the distance between the initial and final horizontal positions for adjustment, and so as the deviation *x* by equation (1). Then, the sample is rotated by 45° and is moved to the measurement position whose horizontal coordinate is corrected by *x*.

The Sample Catcher is controlled by a *LabVIEW* program developed by ourselves, which can be used as a stand-alone system and also as a subroutine that is controlled by the auto-measurement system. The program has a graphical user interface (GUI), and is easily operated by users. The sample image is displayed at the picture box at the left-hand side of the panel. Samples to be manipulated are set by numbers in text boxes, and the sample is manipulated by pressing buttons in the panel. Three different alignment modes can be chosen by a pull-down menu in the panel: auto-, centre- and manual-modes. If the sample shape is readily recognized by the CV-5500, the auto-mode can be used, where a sample is automatically aligned. If a sample is homogeneous and large enough compared with the size of the light [typically 0.5 mm (V) × 5 mm (H) at our beamline], the centre-mode is an appropriate choice, in which the measurement position is set as the centre of the sample holder. If the sample is not recognizable, the operator can adjust the measurement position manually by using the buttons in manual-mode. The measurement mode (transmission/fluorescence) can be selected by another pull-down menu in the panel.

### 2.4. Automatic XAFS measurement system (‘Auto-XAFS’)

The program for automatic XAFS measurement (‘Auto-XAFS’) was also developed by *LabVIEW*, into which the Sample Catcher program and the existing programs for XAFS measurement are integrated as subroutines. This program also has a user-friendly GUI.

The measurement parameters (sample and file names to save, scan ranges of measurement, *etc.*) are indicated in a tabbed pane with eight tabs that include those for ten samples each. Thus the parameters of a total of 80 samples can be set. They can be input directly at the front panel or be loaded from a parameter file (a spreadsheet file of Microsoft Excel) prepared beforehand. The sample alignment mode can be selected by pull-down menus in the rightmost column in the pane. After each measurement, the measurement parameters, the measured data, and the image of the sample captured by the CCD camera are saved.

In XAFS measurement the gains of current amplifiers monitoring the ion current of ion chambers for *I*_0_ and *I* should be adjusted properly for each sample. Precisely speaking, the gains are to be set to the maximum values such that the current amplifiers do not become overloaded through the entire scan range. This adjustment can be made automatically or manually in Auto-XAFS. One of three gain-adjusting modes can be set for each sample, *i.e.* the automatic gain-adjusting, ‘previous’ and manual modes. In the automatic gain-adjusting mode, users can set the gain-adjusting points from one or more of the following, *i.e.* the middle and the end Bragg angles of a scan, and the two extra angles designated for each sample. Then the gains are set to the maximum values that the current amplifiers do not become overloaded at all of the angles selected by users. On the other hand, the gains for the measurement for the previous sample are applied in the ‘previous’ mode, and the gains are set to the values specified for each sample in the manual mode.

Auto-XAFS is applicable for XAFS measurements in both transmission and fluorescence modes.

In transmission mode, the automatic measurement is performed by the following steps: (1) picking up a sample from cassettes, (2) aligning the sample, (3) adjusting the current amplifier gains, (4) measuring the spectrum, and (5) returning the sample to the cassette. Steps (1), (2) and (5) are performed by Sample Catcher, as already described in §2.3. The gains of current amplifiers are adjusted as explained in the previous paragraph.

In fluorescence mode, another step is needed in addition to the steps described for the transmission mode. For the detection of the fluorescent X-rays, 19-SSD is used. The sample is rotated by 45° by the rotary cylinder of Sample Catcher as described in §2.3, and both the incident X-ray and the detector are thus at an angle of 45° with respect to the sample surface. The count rate of incoming X-ray photons to the detector needs to be adjusted to the appropriate value, since too high a count rate causes serious counting loss for counting devices such as solid-state detectors. In Auto-XAFS, this adjustment can be made automatically by varying the horizontal width of the entrance slit and/or the distance between the sample and the detector. The former adjustment controls the flux of the photon onto the sample, and the latter controls the detection efficiency determined by the solid angle of the detector and the attenuation of the fluorescence X-rays by the air. The parameters for this adjustment can be configured at another tabbed pane in the lower part of the front panel.

## 3. Performance

The Sample Catcher can save much time and release users from the tiresome sample-exchanging operation. In the conventional measurement, one has to open the experimental hatch, exchange the measured sample for the next one, align the sample and close the hatch, whenever the XAFS measurement of one sample has been finished. The sample exchange time is reduced to 40–55 s by the introduction of this system, while it costs 90–120 s in the conventional measurements. The inputting time for the measurement conditions which costs 30–60 s in the conventional measurement is reduced to zero. The time for adjusting the gains for current amplifiers is reduced from 60 s to 30 s. Thus Auto-XAFS significantly reduces the total time for XAFS measurement. Furthermore, in the conventional measurement, operators have to exchange and align a sample, adjust the gains of the ion chambers, input the measurement conditions and start the measurement, for each sample. If the measurement time for a sample is short, they have to undertake these procedures every, for example, several minutes. Whereas, in the case of Auto-XAFS, there is nothing to do during the measurement once the measurement conditions for samples are set and auto-measurement is started.

At the same time, the automatic measurement system also improves the accuracy of sample alignment. Actually, the pin-hole scan, the high-precision motorized stages and the high-resolution CCD camera enable us to align samples with an accuracy of 0.15 mm in transmission mode. This accuracy is a big advantage in aligning relatively small samples, while the traditional sample alignment by human eye with a laser-beam is less reliable.

## 4. Current limitation and future prospect of the system

**Figure 4:**
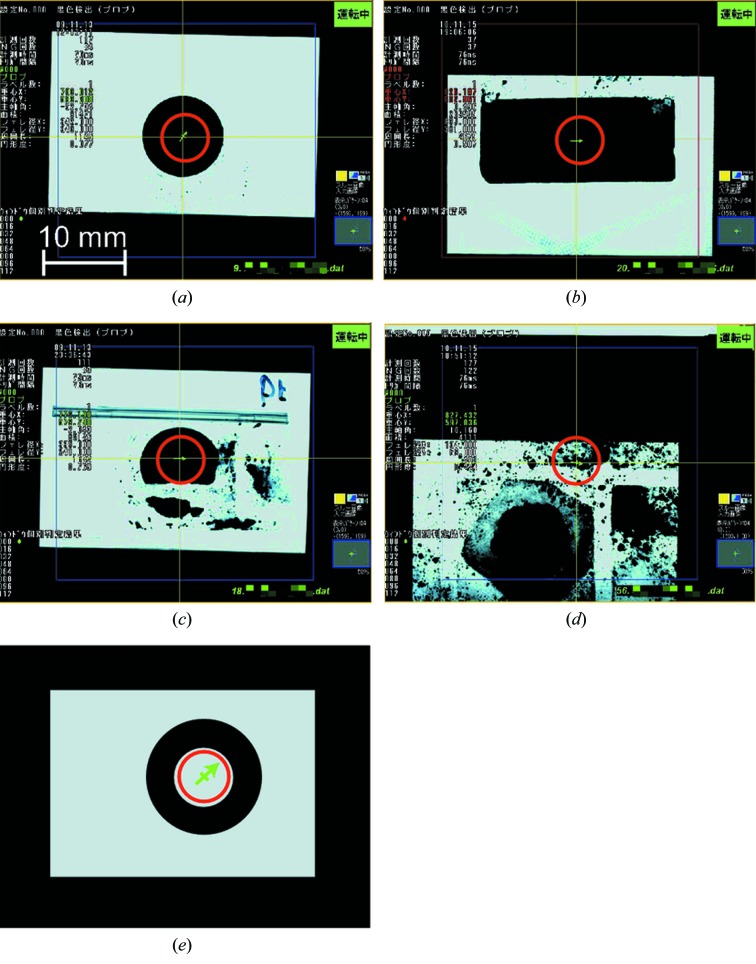
Examples of the sample images captured by CCD camera. The recognized centre-of-gravity in each image is indicated by the midpoint of the green arrow in the red circle. The intersection of yellow lines indicates the reference position. (*a*) A pellet sample of diameter 10 mm pouched in PE film. (*b*) A powder sample pouched in PE film of rectangular shape. (*c*) Broken samples can also be aligned. The samples of (*a*) and (*c*) were measured in transmission mode, and that of (*b*) was measured in fluorescence mode. (*d*) The sample shape is incorrectly recognized because the sample is very dirty. (*e*) If there is a sample with a hole, the centre of gravity of the sample shape is in the hole area with no sample (not the real example).

As implied in §2.1, the centre of gravity of the sample should coincide with the appropriate position for the centre of the X-ray beam in the current version of Auto-XAFS, as in the case shown in Figs. 4(*a*) ▶ (a pellet with 10 mm diameter pouched in PE film measured in transmission mode) and 4(*b*) (a powder sample pouched in PE film measured in fluorescence mode). Broken samples such as Fig. 4(*c*) ▶ can also be aligned, since the largest object in the image is recognized in the system. Otherwise, the sample should be aligned manually in the manual alignment mode. Fig. 4(*d*) ▶ shows one of the examples in which the auto-alignment failed. In this case the recognition of sample shape failed since the sample is very dirty. If there is a sample with a hole [Fig. 4(*e*) ▶, not the real example], the centre of gravity of the sample shape is in the hole area with no sample. The shape of a sample should be carefully examined before the automatic measurement in terms of the availability of the auto-alignment. In addition, users should check the results of the sample recognition stored for individual samples after the measurement to make sure of the correct alignment of the sample.

In the current system the incident beam size cannot be controlled automatically according to the size of the individual sample. In the transmission mode, the slit width is fixed during an auto-measurement run. In the fluorescence mode, users can choose whether to keep the slit width fixed or let it vary according to the signal intensity. The fixed slit width or the adjusting range of the slit width is set by users beforehand. The users should set the slit width so that the width of the footprint of the beam on the sample is within the sample width in all the samples to be measured in a run. The fixed entrance slit is sufficient in most of the cases so far. However, the adjustable slit width will be implemented if there are many requests from the user. It is easy to implement the function to set the slit size for an individual sample manually. Although it is very challenging, it also may be possible to realise the automatic determination of the measurement position and the slit width for an individual sample so that the beam footprint is confined within the sample area, if the function of shape analysis of CV-5500 is utilized.

The adjustment of count rate in fluorescence measurements using slits and/or detector position is not sufficient in some cases, in which selective reduction of coexisting fluorescence and/or scattered radiation is needed. Unfortunately, this part of the adjustment is not automated in our system. In such cases, we should examine manually which filter is appropriate to reduce the coexisting X-rays before starting the auto-measurement run.

The X-ray incidence angle of 45° may not be the best geometry for very thin films, and grazing-incidence geometry is usually more appropriate. Auto-XAFS is not applicable for the grazing-incidence geometry at this moment. However, we are planning to revise the system to be applicable to the fluorescence mode with grazing-incidence geometry. Actually, the special sample holder and the adjusting stages for that purpose are now under design, and the revised system will be available in 2012.

The data processing after the measurements is partly automated so far. When the file of raw data is created after one of the scans, the file is immediately pre-processed automatically. During pre-processing, data rebinning, dead-time correction (in the case of fluorescence mode using 19SSD) and calculation of absorption intensity are performed. The processed data (the rebinned energy and the calculated absorption intensity) are saved in another newly created file. Further data processing (background subtraction, extraction of EXAFS oscillation and Fourier transform) is not automated yet. Users have to load the pre-processed data into their own EXAFS analysis software. However, the automation of further processing is now in progress, and will be realised in the near future.

The systems of Auto-Optics and Auto-XAFS are independent of each other at the moment, but we plan to integrate them to realise the automated measurement including the alignment of the beamline optics, the gases in the ion chambers, gain settings for the current amplifier, sample loading and XAFS measurement. Such a system will be useful if the measurement parameters are already determined before starting the run.

## 5. Summary

We have developed a novel automatic XAFS measurement system which is available in both transmission and fluorescence mode. With this system the XAFS measurements can be performed fully automatically including the sample loading, the gain adjustment for current amplifiers, adjustment of the solid-state detector, and loading of up to a total of 80 samples for batch measurement. An optical adjusting system has also been developed, in which the X-ray optics and the gas flow to the ionization chambers are automatically adjusted. It not only reduces the measurement time and the operators’ labour but also improves reliability of sample alignment, especially for relatively small samples.
